# Effect of dose adjustments on the efficacy and safety of tofacitinib in patients with rheumatoid arthritis: a post hoc analysis of an open-label, long-term extension study (ORAL Sequel)

**DOI:** 10.1007/s10067-021-05908-z

**Published:** 2022-01-01

**Authors:** Ruediger B. Mueller, Hendrik Schulze-Koops, Daniel E. Furst, Stanley B. Cohen, Kenneth Kwok, Lisy Wang, Tim Killeen, Johannes von Kempis

**Affiliations:** 1 Rheumazentrum Ostschweiz, St. Jakobsstr. 20, 9000 St. Gallen, Switzerland; 2 Division of Rheumatology and Immunology, Kantonsspital St. Gallen, St. Gallen, Switzerland; 3 Division of Rheumatology and Clinical Immunology, Department of Internal Medicine IV, Ludwig-Maximilians-University Munich, Munich, Germany; 4 UCLA, Los Angeles, CA USA; 5 University of Washington, Seattle, WA USA; 6 University of Florence, Florence, Italy; 7 Metroplex Clinical Research Center and University of Texas Southwestern Medical Center, Dallas, TX USA; 8 Pfizer Inc, New York, NY USA; 9 Pfizer Inc, Groton, CT USA; 10 Pfizer AG, Zürich, Switzerland

**Keywords:** Dosing, Post hoc analysis, Rheumatoid arthritis, Tofacitinib

## Abstract

**Introduction/objectives:**

We assess the impact of switching versus staying on the same tofacitinib dose on efficacy and safety in patients with rheumatoid arthritis (RA).

**Methods:**

ORAL Sequel was an open-label, long-term extension study of patients with RA receiving tofacitinib 5 or 10 mg BID for up to 9.5 years. Tofacitinib doses could be switched during the study at investigator discretion. In this post hoc analysis, data from ORAL Sequel were stratified into four groups: 5 → 10 mg BID (Dose-up); 5 mg BID (Stay-on 5); 10 → 5 mg BID (Dose-down); and 10 mg BID (Stay-on 10). Efficacy assessments over 12 months included: change from baseline in 4-component Disease Activity Score in 28 joints, erythrocyte sedimentation rate (DAS28), and DAS28 minimum clinically important difference, remission, and low disease activity (LDA) rates. Safety was assessed for the study duration.

**Results:**

Generally, DAS28 improvements and minimum clinically important difference rates were significantly greater (*p* < 0.05) in Dose-up versus Stay-on 5 up to month 12. DAS28 remission rates were significantly greater in Dose-up versus Stay-on 5 at month 12. Change from baseline in DAS28 was similar in Dose-down and Stay-on 10. No significant differences in DAS28 LDA rates were observed between groups. Safety data were similar overall across the four groups.

**Conclusion:**

In patients with RA receiving open-label tofacitinib, this analysis found that some benefited from increasing dose from 5 to 10 mg BID and did not find that reducing dose from 10 to 5 mg BID affected efficacy or that dose switching in either direction affected safety.

**Study registration:**

ClinicalTrials.gov number NCT00413699. Registered December 20, 2006. https://clinicaltrials.gov/ct2/show/NCT00413699

**Table:** 

**Key Points** *• This post hoc analysis of data from the long-term extension study, ORAL Sequel, assessed the impact of dose switching between tofacitinib 5 and 10 mg twice daily (BID), at the investigator’s discretion, on efficacy and safety in patients with rheumatoid arthritis (RA).* *• Dosing up from tofacitinib 5 to 10 mg BID was associated with improved efficacy up to 12 months versus staying on 5 mg BID, and dosing down from 10 to 5 mg BID was not generally associated with a significant loss of efficacy.* *• Safety outcomes were generally consistent across dose groups and did not change markedly after switching dose in either direction.* *• These findings can help to inform physicians on what may be expected in terms of efficacy and safety when adjusting tofacitinib dose according to clinical need. The recommended tofacitinib dosage for the treatment of RA in most jurisdictions is 5 mg BID.*

**Supplementary Information:**

The online version contains supplementary material available at 10.1007/s10067-021-05908-z.

## Background

Rheumatoid arthritis (RA) is a chronic inflammatory disease, which can lead to joint destruction, disability, and decreased quality of life [1, 2]. The American College of Rheumatology (ACR) and European Alliance of Associations for Rheumatology (EULAR) advise a treat-to-target approach for RA, with treatment goals of remission or low disease activity (LDA) [3, 4]. EULAR specifically recommends aiming for a rapid attainment of treatment goals within 3–6 months [4]. Over the past decade, Janus kinase (JAK) inhibitors have emerged as a valuable option for patients whose RA is not fully controlled by conventional synthetic disease-modifying antirheumatic drugs (csDMARDs) [5–7].

Tofacitinib is an oral JAK inhibitor for the treatment of RA. The efficacy and safety of tofacitinib 5 and 10 mg twice daily (BID) administered as monotherapy or with csDMARDs, mainly methotrexate (MTX), in patients with moderately to severely active RA, have been demonstrated in phase 2 [8–12], phase 3 [13–19], and phase 3b/4 [20] studies of up to 24 months’ duration, and in long-term extension (LTE) studies with up to 114 months’ observation [21–23].

A post hoc analysis of patients with RA who were required to switch from 5 to 10 mg BID or from 10 to 5 mg BID on entry to tofacitinib LTE studies generally showed no significant differences, in terms of efficacy or safety, between those who switched dose up or down [24]. However, such per-protocol switches are not directly informative for clinical decision-making in daily practice, where treating physicians typically adjust dose in response to disease activity, specific patient characteristics, and disease presentation in the context of patient factors. As such, it remains unclear as to what physicians should expect in terms of a loss or gain of efficacy when adjusting dose based on clinical need, and whether dose switches are associated with particular adverse events (AEs).

ORAL Sequel (NCT00413699) was a global, multicenter, open-label, LTE study, which primarily assessed the long-term safety and tolerability of tofacitinib 5 and 10 mg BID [22]. The safety profile over 114 months remained consistent with that observed in phase 2 and 3 tofacitinib studies [22]. Additionally, sustained efficacy of tofacitinib 5 and 10 mg BID was demonstrated up to 96 months [22].

In this post hoc analysis of ORAL Sequel, we aimed to characterize efficacy and safety of tofacitinib following dose switches initiated at investigator discretion in both directions between 5 and 10 mg BID, versus patients who stayed on the same dosage throughout study follow-up. It is important to note that the recommended tofacitinib dosage for the treatment of RA in most jurisdictions is 5 mg BID.

## Methods

### Study design

This post hoc analysis included data from ORAL Sequel. Patients with RA who participated in phase 1, 2, or 3 index studies of tofacitinib were eligible to enter this LTE study. Eligibility criteria for entry into the index studies varied and included patients with prior inadequate response to csDMARDs or biologic DMARDs (bDMARDs), and patients who were MTX-naïve. All patients had a diagnosis of RA and were required to meet the ACR 1987 Revised criteria in order to be eligible for the index studies (except Study A3921237 [NCT02147587], in which patients were required to meet the 2010 ACR/EULAR criteria with an RA score of ≥ 6). Most patients received tofacitinib 5 or 10 mg BID in the index studies. Further details of the study design of ORAL Sequel have been previously reported [22].

The study was conducted in accordance with  International Council for Harmonization Guidelines for Good Clinical Practice and the Declaration of Helsinki. The study protocol was reviewed and approved by the Institutional Review Boards and/or an independent ethics committee at each study center, and all patients provided written informed consent.

### Study treatment

On entry to ORAL Sequel, patients received tofacitinib 5 or 10 mg BID (as mandated by the protocol). Patients received tofacitinib as monotherapy or with stable background RA therapy, which included csDMARDs and/or glucocorticoids (≤ 10-mg/day prednisone or equivalent) but not bDMARDs.

Most patients from phase 2 index studies initiated ORAL Sequel with tofacitinib 5 mg BID, and most patients from phase 3 index studies initiated with tofacitinib 10 mg BID. Patients in China were required, per-protocol, to receive tofacitinib 5 mg BID on entry to ORAL Sequel. Patients in the Republic of Korea newly enrolled from August 2014 onwards were required to receive tofacitinib 5 mg BID on entry to ORAL Sequel. Patients in Japan completing phase 2 and phase 3 index studies were enrolled in a separate LTE study (A3921041; NCT00661661) [21] and were not included in this analysis.

During ORAL Sequel, tofacitinib dose adjustments could be made at the investigator’s discretion (Supplementary Table 1). The reasons for dose adjustment were not recorded. Patients in the Republic of Korea receiving tofacitinib 10 mg BID prior to August 2014 were required to dose down to 5 mg BID to remain in the study; these patients were excluded from this analysis because the dose adjustment was protocol-mandated rather than at the investigator’s discretion.

### Analysis population and analysis groups

Patients were analyzed in four groups: Dose-up and Dose-down (collectively dose-switch) and Stay-on 5 and Stay-on 10. Dose-up patients were those who received tofacitinib 5 mg BID as their initial LTE dose and then changed to 10 mg BID. Dose-down patients were those who received tofacitinib 10 mg BID as their initial LTE dose and then changed to 5 mg BID. In order to include ≥ 2 assessments and exclude patients with lengthy gaps in therapy, Dose-up and Dose-down patients must have received their initial LTE dose for ≥ 81 days and have had a ≤ 14-day dose gap between initial and new doses. Stay-on 5 and Stay-on 10 patients were those who received tofacitinib 5 or 10 mg BID, respectively, as their initial LTE dose for ≥ 81 days, and remained on it for the entire duration of their LTE study participation.

### Assessments

Efficacy endpoints included changes from baseline in 4-component Disease Activity Score in 28 joints, erythrocyte sedimentation rate (DAS28), Health Assessment Questionnaire-Disability Index (HAQ-DI), Clinical Disease Activity Index (CDAI), and Simplified Disease Activity Index (SDAI). Rates of achieving minimum clinically important difference (MCID) in DAS28 (decrease in DAS28 of ≥ 1.2) [25], remission (DAS28 scores < 2.6, CDAI scores ≤ 2.8, SDAI scores ≤ 3.3), and DAS28 LDA (scores ≤ 3.2) were evaluated. Safety endpoints included AEs, discontinuations due to AEs, serious AEs, and AEs of special interest.

### Statistical analyses

Data were analyzed from the final LTE study data cut (March 02, 2017). The analysis baseline for the Dose-switch groups was defined as the last observation before or on the day of the (first) dose switch during the LTE; only patients with ≥ 1 efficacy observation after switching dose were included in the efficacy analysis. Analysis baseline for the Stay-on groups was defined as the LTE month 3 visit observation; only patients with ≥ 1 efficacy observation after the month 3 visit were included in the efficacy analysis.

In the efficacy analyses, for dose-switch patients with a single switch, data were included up to 12 months after baseline. For those with multiple dose switches during the LTE period, only data up to the second switch or 12 months after the first switch, whichever was earlier, were used for the analyses. For Stay-on patients, LTE data from the first 12 months after the month 3 visit were included. Changes in efficacy endpoints from baseline were compared between the Dose-up and Stay-on 5 groups, and separately between the Dose-down and Stay-on 10 groups. Statistical comparisons were performed on observed values using repeated measures mixed models, controlling for geographical region, tofacitinib exposure prior to the analysis baseline, RA disease duration, and baseline efficacy endpoint score. For DAS28 MCID, remission, and LDA rates, and CDAI remission rates and SDAI remission rates, comparisons were performed using Generalized Estimating Equations repeated measure models, adjusting for geographical region, tofacitinib exposure prior to analysis baseline, RA disease duration, and baseline DAS28, CDAI, or SDAI score. Missing data were not imputed (proportions of patients with missing data stratified by efficacy outcome are presented in Supplementary Table 2). *p* values were not adjusted for multiplicity, due to the exploratory nature of the analyses.

In the safety analyses, exposure-adjusted event rates (EAERs; patients with events per 100 patient-years) were calculated for Months 0–3, 3–6, 6–12, and > 12 for the most common AEs by preferred term, AEs in the system organ class (SOC) of investigations, and selected investigations by higher-level group term. The most common AEs were defined as those for which EAERs were > 6 in months 0–3, 3–6, 6–12, or > 12, in any of the four dose groups. AEs were assigned to Dose-up and Dose-down groups according to the first switch that a patient experienced, even if patients switched doses again. AEs included new events occurring during the LTE study period or worsening of pre-existing AEs that occurred during the index or LTE study periods. The percentage of patients who experienced serious AEs, and incidence rates (unique patients with events per 100 patient-years) for safety events of special interest, were calculated. In patients who experienced multiple dose switches, safety data were not included beyond the second dose switch. However, a sensitivity analysis was performed, in which these data were included.

## Results

### Patients

During ORAL Sequel, 1049 (23.4%) of 4481 patients switched dose at least once, 218 (4.9%) switched dose twice, and 90 (2.0%) switched dose more than twice. In this post hoc analysis of 1037 patients commencing on tofacitinib 5 mg BID, 280 (27.0%) had their dose increased by the investigator to 10 mg BID (Dose-up). Of 2916 patients commencing on tofacitinib 10 mg BID, 476 (16.3%) had their dose reduced by the investigator to 5 mg BID (Dose-down); 63 patients in the Republic of Korea who were required to reduce their dose were excluded from the analysis. On average, patients in the Dose-down group were treated with their initial tofacitinib dose slightly longer than patients in the Dose-up group (mean [95% CI]: 800.7 [752.4, 849.0] vs 634.1 [563.2, 705.0] days); however, the 95% CIs were large and close together, so these data should be interpreted with caution. Patient demographics were generally similar across treatment groups (Table 1). However, a greater proportion of patients in the Dose-down group were Asian, compared with the Stay-on 10 group (18.9% vs 10.9%). Baseline disease activity indices and concomitant therapy use rates did not vary greatly between groups, but patients in the Dose-up group had higher disease activity and were more likely to have been receiving tofacitinib as monotherapy at baseline, than that of those who stayed on 5 mg BID (38.2% vs 28.5%; Table 1).

**Table 1:** Patient demographics and baseline^a^ disease characteristics

Characteristic	Dose-up (*N* = 280)	Stay-on 5 (*N* = 757)	Dose-down (*N* = 476)	Stay-on 10 (*N* = 2440)
Age (years), mean (range)	51.7 (20.0–75.0)	53.6 (18.0–81.0)	55.5 (20.0–86.0)	53.0 (18.0–85.0)
Female, *n* (%)	231 (82.5)	630 (83.2)	395 (83.0)	1980 (81.1)
Race, *n* (%)				
White	191 (68.2)	489 (64.6)	325 (68.3)	1842 (75.5)
Black	7 (2.5)	18 (2.4)	17 (3.6)	76 (3.1)
Asian	53 (18.9)	157 (20.7)	90 (18.9)	266 (10.9)
Other	29 (10.4)	93 (12.3)	44 (9.2)	256 (10.5)
RA duration (years), mean (range)	8.0 (0.7–49.5)	9.7 (0.7–46.5)	9.7 (0.6–50.6)	8.7 (0.3–55.5)
DAS28, mean (SD)	4.5 (1.6)	3.7 (1.3)	3.4 (1.2)	3.6 (1.3)
HAQ-DI, mean (SD)	1.0 (0.7)	0.8 (0.7)	0.7 (0.7)	0.8 (0.7)
CDAI, mean (SD)	18.1 (14.0)	11.4 (9.8)	8.5 (8.6)	10.3 (9.9)
SDAI, mean (SD)	18.9 (14.4)	11.8 (9.8)	9.0 (8.8)	10.8 (10.0)
RF + , *n* (%)	200 (71.4)	538 (71.1)	333 (70.0)	1636 (67.0)
aCCP + , *n* (%)^b^	101 (36.1)	161 (21.3)	334 (70.2)	1701 (69.7)
Tofacitinib as monotherapy, *n* (%)	107 (38.2)	216 (28.5)	163 (34.2)	802 (32.9)
Corticosteroid use, *n* (%)	148 (52.9)	387 (51.1)	218 (45.8)	1231 (50.5)

^a^All values are for LTE baseline, except DAS28, HAQ-DI, CDAI, and SDAI, which are values at analysis baseline. For Dose-switch groups, analysis baseline was defined as the last observation before or on the day of the first dose switch. For Stay-on groups, analysis baseline was defined as month 3 LTE visit

^b^Data were only available for 133 (48%) patients in the Dose-up group, 189 (25%) patients in the Stay-on 5 group, 447 (94%) patients in the Dose-down group, and 2236 (92%) patients in the Stay-on 10 group

*aCCP* + anti-cyclic citrullinated peptide positive, *CDAI* Clinical Disease Activity Index, *DAS28* 4-component disease activity score in 28 joints, erythrocyte sedimentation rate, *HAQ-DI* Health Assessment Questionnaire-Disability Index, *LTE* long-term extension, *N* number of patients in treatment group, *n* number of patients with characteristic, *RA* rheumatoid arthritis, *RF* + rheumatoid factor positive; *SD* standard deviation, *SDAI* Simplified Disease Activity Index

### Efficacy

**Fig. 1:**
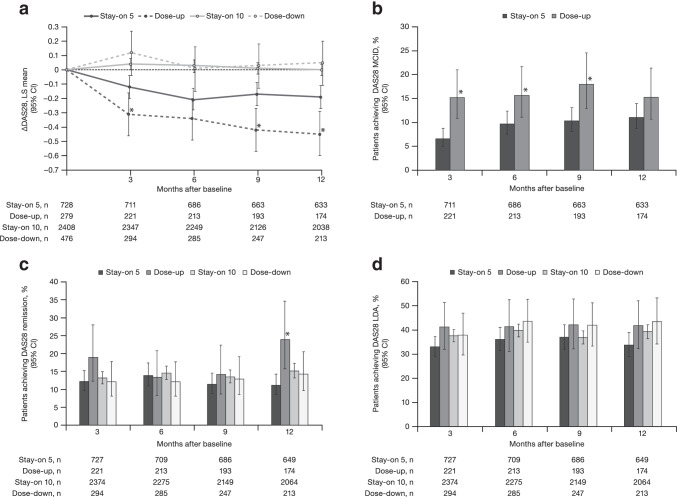
**a** ΔDAS28, **b** DAS28 MCID,^a^ **c** DAS28 remission, and **d** DAS28 LDA over 12 months. *p < 0.05, Dose-up vs Stay-on 5. ^a^Data are presented only for Dose-up and Stay-on 5 as MCID is not relevant in the context of dosing down *Δ* change from baseline, *CI* confidence interval, *DAS28* 4-component disease activity score in 28 joints, erythrocyte sedimentation rate, *LDA* low disease activity, *LS* least squares, *MCID* minimum clinically important difference, *n* number of evaluable patients

A significantly greater reduction in DAS28 score from baseline was observed in the Dose-up group versus the Stay-on 5 group at months 3, 9, and 12 (Fig. 1a). In contrast, no significant difference was observed in change from baseline in DAS28 between the Dose-down and Stay-on 10 groups (Fig. 1a). Rates of achieving MCID in DAS28 were significantly higher in the Dose-up group versus the Stay-on 5 group up to month 9 (Fig. 1b). DAS28 remission rates were significantly higher in the Dose-up group versus the Stay-on 5 group at month 12 (Fig. 1c). There were no significant differences in DAS28 remission rates between the Dose-down group and the Stay-on 10 group (Fig. 1c). There were also no significant differences in LDA rates between the Dose-up group and the Stay-on 5 group, nor between the Dose-down group and the Stay-on 10 group (Fig. 1d).

**Fig. 2:**
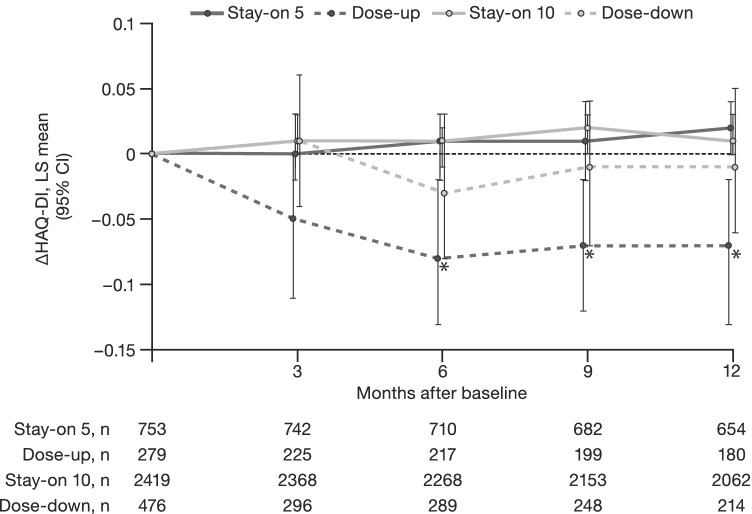
ΔHAQ-DI over 12 months. *p < 0.05, Dose-up vs Stay-on 5 *Δ* change from baseline, *CI* confidence interval, *HAQ-DI* Health Assessment Questionnaire-Disability Index, *LS* least squares, *n* number of evaluable patients

In the Dose-up group, a significant improvement in HAQ-DI scores from baseline versus the Stay-on 5 group was observed from month 6 onwards (Fig. 2). No significant differences were observed between the Dose-down and Stay-on 10 groups with respect to change from baseline in HAQ-DI (Fig. 2).

Similarly, significant improvements in CDAI and SDAI scores from baseline were observed in the Dose-up group versus the Stay-on 5 group from month 9 onwards (Supplementary Fig. 1). CDAI and SDAI remission rates were also significantly greater in the Dose-up group versus the Stay-on 5 group at month 9 (Supplementary Fig. 1). There were no significant differences in change from baseline in CDAI or SDAI, nor in CDAI or SDAI remission rates, between the Dose-down group and the Stay-on 10 group (Supplementary Fig. 1).

### Safety

The percentage of patients who experienced AEs or serious AEs was broadly similar across the four dose groups (Table 2). However, in the Dose-down group versus the Stay-on 10 group, a higher percentage of patients experienced AEs at months 0–3 (39.1% vs 33.4%), and a lower percentage of patients experienced AEs at months > 12 (63.4% vs 83.0%) (Table 2). Patients in the Dose-switch groups were less likely to discontinue treatment due to AEs in months > 12 than patients in the Stay-on groups (Table 2).

**Table 2:** Summary of all-causality AEs

AEs by selected time points^a^	Dose-up^b^ (*N* = 280)		Stay-on 5^c^ (*N* = 757)		Dose-down^b^ (*N* = 476)		Stay-on 10^c^ (*N* = 2440)	
	Months 0–3	Months > 12	Months 0–3	Months > 12	Months 0–3	Months > 12	Months 0–3	Months > 12
Evaluable for AEs, *n*	280	186	757	663	476	213	2440	2072
Total exposure, years	67	400	184	2533	103	277	594	5492
Patients with AEs, *n* (%)	93 (33.2)	142 (76.3)	210 (27.7)	533 (80.4)	186 (39.1)	135 (63.4)	816 (33.4)	1719 (83.0)
Patients who discontinued due to AEs, *n* (%)	5 (1.8)	20 (10.8)	10 (1.3)	161 (24.3)	19 (4.0)	20 (9.4)	43 (1.8)	384 (18.5)
Most common AEs by preferred term, *n* (EAER per 100 patient-years)^d^								
Blood creatine phosphokinase increased	2 (2.99)	11 (2.75)	3 (1.62)	35 (1.38)	7 (6.78)	8 (2.88)	13 (2.19)	96 (1.74)
Bronchitis	5 (7.49)	18 (4.50)	4 (2.16)	74 (2.92)	4 (3.87)	12 (4.32)	32 (5.39)	217 (3.95)
Nasopharyngitis	3 (4.49)	12 (3.00)	10 (5.42)	67 (2.64)	10 (9.68)	12 (4.32)	28 (4.71)	218 (3.96)
Rheumatoid arthritis^e^	8 (11.99)	20 (5.00)	3 (1.62)	42 (1.65)	11 (10.65)	16 (5.76)	18 (3.03)	142 (2.58)
Upper respiratory tract infection	2 (2.99)	15 (3.75)	10 (5.42)	106 (4.18)	10 (9.68)	20 (7.20)	55 (9.26)	289 (5.26)
Urinary tract infection	5 (7.49)	15 (3.75)	12 (6.50)	73 (2.88)	6 (5.81)	10 (3.60)	39 (6.57)	220 (4.00)
Investigations (SOC), *n*	11	37	23	178	29	26	72	400
(EAER per 100 patient-years)	(16.49)	(9.25)	(12.46)	(7.02)	(28.09)	(9.37)	(12.13)	(7.28)
Selected investigations by higher-level group term, *n* (EAER per 100 patient-years)								
Hepatobiliary investigations	4 (5.99)	13 (3.25)	10 (5.42)	48 (1.89)	9 (8.71)	11 (3.96)	22 (3.70)	113 (2.05)
Renal and urinary tract investigations and urinalyses	2 (2.99)	8 (2.00)	2 (1.08)	42 (1.65)	8 (7.75)	3 (1.08)	4 (0.67)	40 (0.72)
AEs in overall period^f^								
Patients with serious AEs, *n* (%) [EAER per 100 patient-years]	52 (18.6) [8.4]		230 (30.4) [7.1]		58 (12.2) [10.1]		713 (29.2) [9.2]	
AEs of special interest, IR per 100 patient-years (95% CI) [*n* (%)]								
Herpes zoster	1.75 (0.87–3.13) [11 (3.9)]		2.60 (2.05–3.23) [79 (10.4)]		2.86 (1.67–4.58) [17 (3.6)]		3.68 (3.26–4.14) [274 (11.2)]	
Serious infections	2.38 (1.33–3.92) [15 (5.4)]		1.99 (1.53–2.53) [65 (8.6)]		2.81 (1.64–4.50) [17 (3.6)]		2.84 (2.48–3.23) [225 (9.2)]	
Deep vein thrombosis	0.00 (0.00–0.58) [0 (0.0)]		0.09 (0.02–0.27) [3 (0.4)]		0.00 (0.00–0.61) [0 (0.0)]		0.20 (0.11–0.33) [16 (0.7)]	
Pulmonary embolism	0.00 (0.00–0.58) [0 (0.0)]		0.06 (0.01–0.22) [2 (0.3)]		0.16 (0.00–0.92) [1 (0.2)]		0.15 (0.08–0.26) [12 (0.5)]	

^a^The reporting period (0–3 months or > 12 months) is based on analysis baseline as 0 months; these reporting periods were selected to represent short and long latency events, respectively

^b^For Dose-switch groups, analysis baseline was defined as the day of the (first) dose switch

^c^For Stay-on groups, analysis baseline was defined as LTE month 3 visit

^d^AEs are presented for each dose group where EAER > 6 in any dose group at months 0–3 or > 12

^e^All AEs coded as “rheumatoid arthritis” in ORAL Sequel, except one, indicated worsening of rheumatoid arthritis

^f^Overall period defined as all data after analysis baseline for Stay-on groups, or all data after analysis baseline up to second dose switch for Dose-switch groups

*AE* adverse event, *CI* confidence interval, *EAER* exposure-adjusted event rate per 100 patient-years, *IR* incidence rate, *LTE* long-term extension, *N* number of patients in treatment group, *n* number of patients with event, *SOC* system organ class

In months 0–3 and months > 12, EAERs (per 100 patient-years) of the most common AEs by preferred term were generally similar between the Dose-up and Stay-on 5 groups. However, the EAER of RA as an AE was higher in the Dose-up group than the Stay-on 5 group (11.99 vs 1.62 in months 0–3; 5.00 vs 1.65 in months > 12).

The EAERs of the most common AEs by preferred term were generally numerically higher in the Dose-down group than the Stay-on 10 group in months 0–3, although rates were similar between these groups in months > 12. Difference observed between these groups in months 0–3 included elevated blood creatine phosphokinase (EAER: 6.78 vs 2.19).

AEs in the SOC of investigations had a notably higher EAER in the Dose-down group versus the Stay-on 10 group at months 0–3. In particular, there were increased EAERs for hepatobiliary investigations and renal and urinary tract investigations and urinalyses, in the Dose-down group versus the Stay-on 10 group (Table 2). There were not such notable differences in the Dose-up group versus the Stay-on 5 group. Overall, the rates of investigations by preferred term were low.

Similar overall trends in AEs were observed in months 3–6 and 6–12 (Supplementary Table 3). Furthermore, a sensitivity analysis including data beyond the second dose switch for patients who experienced multiple dose switches revealed slightly higher AE rates in general, versus the rates when data were analyzed only up to the second dose switch (Supplementary Table 4).

Incidence rates of herpes zoster (HZ), serious infections (SIs), deep vein thrombosis (DVT), and pulmonary embolism (PE) did not differ significantly (95% CIs overlapped) in the Dose-up group versus the Stay-on 5 group or in the Dose-down group versus the Stay-on 10 group (Table 2).

While the analysis was not designed to compare the Stay-on 5 and Stay-on 10 groups, it is notable that the incidence rates of HZ and SI were numerically higher in the Stay-on 10 group versus the Stay-on 5 group (Table 2). Incidence rates of DVT and PE were also numerically higher in the Stay-on 10 group, but the 95% CIs overlapped substantially.

## Discussion

This post hoc analysis of data from ORAL Sequel assessed the impact of changes in tofacitinib dose on efficacy and safety, which were at the investigators’ discretion and based on clinical decisions regarding response, AEs, and laboratory abnormalities. Data from patients with RA who increased or decreased their tofacitinib dose from their baseline dose (5 or 10 mg BID) were compared with patients who stayed on their baseline dose.

Many DMARDs offer prescribers flexibility with different dosing regimens, permitting a tailored approach by means of scaling up treatment to meet therapeutic targets or to treat flares in RA, or dosing down in response to AEs or remission [3, 4]. In clinical practice, Dose-up strategies with csDMARDs can result in improved outcomes for patients with RA [26, 27], suggesting a rationale for this approach. The judicious dosing down of therapies in the context of sustained (≥ 6 months) remission or LDA can be part of the therapeutic strategy, according to the most recent EULAR guidelines for the management of RA [4].

In this analysis, dosing up from tofacitinib 5 to 10 mg BID was associated with improved efficacy up to 12 months compared with staying on 5 mg BID. This was primarily demonstrated by DAS28 improvements and DAS28 MCID and remission rates, and supported by HAQ-DI, CDAI, and SDAI improvements, and CDAI and SDAI remission rates. Importantly, patients dosing down from 10 to 5 mg BID generally did not significantly differ in terms of their disease activity, versus those remaining on 10 mg BID.

An improvement in DAS28 in the Stay-on 5 group was observed during the first few months analyzed (Fig. 1a). This may be because patients receiving tofacitinib 5 mg BID had less cumulative tofacitinib exposure and were still demonstrating clinical improvements beyond LTE initiation, while patients receiving tofacitinib 10 mg BID appeared to have reached their treatment plateau, as an improvement in DAS28 was not observed. In addition, some patients had only completed relatively short index studies prior to entering ORAL Sequel, which may have prolonged the required time to symptom resolution.

The safety findings were generally consistent across dose groups, and also with the overall ORAL Sequel population [22]. Despite the higher dose of tofacitinib received by patients, dosing up was not necessarily associated with an increase in AEs. The overall rates of AEs, and the rates of AEs previously associated with JAK inhibition, such as HZ, were similar to those previously reported [28] and did not change markedly after switching dose in either direction. Rates of AEs were higher in the Dose-down group than the Stay-on 10 group in months 0–3, although this may be due to confounding by indication; i.e., ongoing AEs may have been the reason for dosing down. In months > 12, rates of AEs were lower in the Dose-down group versus the Stay-on 10 group. Interestingly, patients in both Dose-switch groups were less likely to discontinue tofacitinib treatment due to AEs after 12 months, compared with patients in the Stay-on groups, suggesting that optimizing dosage may have increased drug survival in this study, although patients who tolerated tofacitinib 5 mg BID well were probably more likely to be selected for dose increase. The relatively high frequency of laboratory investigations in both Dose-switch groups, particularly hepatobiliary and renal tests (tofacitinib is excreted both hepatically and renally [29]), are compatible with the practice of routinely ordering tests after dose adjustment. Nevertheless, the highest rate of investigations was seen in the Dose-down group, suggesting that abnormalities in these organ systems may have driven a switch from tofacitinib 10 to 5 mg BID in some patients, although this is speculative.

While this analysis did not intend to draw comparisons between patients who stayed on tofacitinib 5 or 10 mg BID, incidence rates of HZ, SI, DVT, and PE were numerically, but not significantly, higher in the Stay-on 10 group versus the Stay-on 5 group. No significant differences in the incidence rates of DVT, PE, HZ, or SI were observed between the Dose-up and Stay-on 5 groups and between the Dose-down and Stay-on 10 groups, although event and patient numbers were low in the Dose-switch groups. The DVT and PE rates were similar to the incidence rates (patients with events per 100 patient-years [95% CI]) in the overall tofacitinib population in ORAL Sequel (*n* = 4481): 0.1 (0.1 to 0.2) and 0.1 (0.1 to 0.2) for DVT and PE, respectively [22]. Study A3921133 is a recently completed, open-label, endpoint-driven study evaluating the safety of tofacitinib 5 and 10 mg BID compared with tumor necrosis factor inhibitors (TNFi) in patients with RA. Patients had to be 50 years of age or older, have at least one additional cardiovascular risk factor, and be on a stable dose of MTX to be eligible for enrollment. The results from Study A3921133 showed an increased rate for tofacitinib relative to TNFi regarding venous thromboembolism (VTE), major adverse cardiovascular events (MACE), and malignancies [30, 31]. Subsequently, based on the information from Study A392113 and consideration of information pertaining to VTE for other JAK inhibitors, Pfizer has determined that VTE is an important identified risk for treatment with tofacitinib. Moreover, MACE and malignancies are important potential risks. Given that the underlying mechanism(s) for these AEs remain unknown, the effect of JAK inhibitors on cardiovascular disease and malignancy risk requires further research. A separate, comprehensive manuscript reporting DVT and PE events across the tofacitinib clinical program has recently been published [32]. Since ORAL Sequel was completed in 2016 prior to these findings, it is unlikely that dose-switching behavior in this analysis was influenced by concerns about potential VTE with the higher dose. Importantly, these findings, together with the results of the current analysis, provide additional insight on the use of tofacitinib that can lead to better informed treatment decisions.

In the sensitivity analysis, which included safety data for patients who experienced multiple dose switches, the rates of AEs were generally slightly higher compared with those when data were analyzed only up to the second dose switch. These observations may be due to confounding by indication, as the reason for multiple dose switches may have been because the patients experienced AEs.

While rates of concomitant csDMARD use at baseline were similar in the Dose-down and Stay-on 10 groups, a higher proportion of patients in the Dose-up group were on tofacitinib monotherapy at the point of dose adjustment. This may reflect a tendency by investigators to preferentially increase the dose of csDMARD rather than tofacitinib when identifying a need to escalate therapy. As this is not an option in patients on monotherapy (although a csDMARD could, of course, be added, if not contraindicated), investigators may have been more likely to increase tofacitinib dose in these patients. Alternatively, investigators may have increased the dose of tofacitinib prophylactically when the clinical situation required the withdrawal of csDMARD therapy. However, these remain speculations, as the reasons for dose adjustment were not recorded.

The proportion of patients who were Asian was notably lower in the Stay-on 10 group versus other groups, likely because Chinese patients were protocol-mandated to initiate ORAL Sequel on 5 mg BID. This underrepresentation did not, however, extend to the Dose-down group, suggesting that those Asian patients receiving 10 mg BID were more likely to have their dose reduced than patients of other races. The increased risk of HZ in Japanese and Korean patients receiving tofacitinib was well known while ORAL Sequel was ongoing [33], and generalization of this perceived risk more broadly to Asian patients may have motivated physicians to dose down more readily in this group.

The effect of dose adjustment can be studied in a clinical trial setting by randomizing patients to dose escalation or reduction. For example, in a randomized, blinded substudy within a phase 3 study of baricitinib in patients with RA, a significantly greater proportion of patients who dosed down from 4 to 2-mg once-daily lost disease control, versus those who stayed on 4-mg once-daily (CDAI remission [≤ 2.8]: 61% vs 76%, respectively) [34]. However, this randomized study design bears little resemblance to the reality encountered by prescribers, who generally adjust doses based on the clinical situation and patient preferences. A key strength of the analysis presented here for tofacitinib is that it accounts for the physician’s role in selecting patients for dose adjustment.

Limitations of this analysis must also be considered. ORAL Sequel was not designed to assess differences in efficacy and safety between the treatment groups analyzed herein, nor to assess the reasons for dose adjustments, which cannot be inferred (e.g., from blood results or safety events) without an unacceptable degree of inaccuracy and bias. This analysis is therefore limited by its post hoc nature, the open-label study design, and the low patient numbers in some groups. The fact that data were used from an LTE study meant that this patient population had already shown tolerability for tofacitinib and drug retention in the index studies. Furthermore, whether patients received 5 or 10 mg BID at LTE entry depended on their initial randomization or jurisdictional requirements, and, as such, some patients may have been switched because they or their physician believed that they were on an inappropriate dose. Therefore, it cannot be assumed that all dose-switch decisions in this cohort were made purely for efficacy or safety reasons. It is, however, likely that disease activity would be a key driver. As such, it would be expected that disease activity at the time of switching would be high in patients who were switched from tofacitinib 5 to 10 mg BID, and low in patients who were switched from tofacitinib 10 to 5 mg BID. Although differences in disease activity at baseline (last observation before or on the day of the first dose switch) were adjusted for in these analyses, a potential bias remains when analyzing data at the time of switching. Although patients in the Dose-down group were, on average, treated with their initial tofacitinib dose slightly longer than patients in the Dose-up group, the exact timing of dose switch is difficult to determine, as patients may have switched their dose between visits. Finally, these analyses were not adjusted for prior csDMARD or bDMARD use/inadequate response.

## Conclusions

To conclude, this post hoc analysis of data from ORAL Sequel revealed that increasing tofacitinib dose from 5 to 10 mg BID in an open-label clinical trial setting appeared to improve tofacitinib efficacy in treating signs and symptoms of RA, but reducing the dose from 10 to 5 mg BID was not generally associated with a significant loss of efficacy. Dose switching in either direction did not appear to affect safety. Numerically higher rates of HZ, SI, DVT, and PE were observed in patients who stayed on tofacitinib 10 mg BID versus those who stayed on 5 mg BID. It is important to note that the recommended tofacitinib dosage for the treatment of RA in the majority of jurisdictions is 5 mg BID.

## Supplementary Information

Below is the link to the electronic supplementary material.

Supplementary file1 (PDF 484 KB)

## Data Availability

Upon request, and subject to review, Pfizer will provide the data that support the findings of this study. Subject to certain criteria, conditions and exceptions, Pfizer may also provide access to the related individual de-identified participant data. See https://www.pfizer.com/science/clinical-trials/trial-data-and-results for more information.

## Abbreviations

aCCP +: Anti-cyclic citrullinated peptide positive
ACR: American College of Rheumatology
AE: Adverse event
bDMARD: Biologic DMARD
BID: Twice daily
CDAI: Clinical Disease Activity Index
CI: Confidence interval
csDMARD: Conventional synthetic DMARD
DAS28: 4-component Disease Activity Score in 28 joints, erythrocyte sedimentation rate
DMARD: Disease-modifying antirheumatic drug
DVT: Deep vein thrombosis
EAER: Exposure-adjusted event rate
EULAR: European Alliance of Associations for Rheumatology
HAQ-DI: Health Assessment Questionnaire-Disability Index
HZ: Herpes zoster
IR: Incidence rate
JAK: Janus kinase
LDA: Low disease activity
LS: Least squares
LTE: Long-term extension
MACE: Major adverse cardiovascular events
MCID: Minimum clinically important difference
MTX: Methotrexate
PE: Pulmonary embolism
RA: Rheumatoid arthritis
RF +: Rheumatoid factor positive
SD: Standard deviation
SDAI: Simplified Disease Activity Index
SI: Serious infection
SOC: System organ class
TNFi: Tumor necrosis factor inhibitor
VTE: Venous thromboembolism
