# Supplementary material for: Effect of dose adjustments on the efficacy and safety of tofacitinib in patients with rheumatoid arthritis: a post hoc analysis of an open-label, long-term extension study (ORAL Sequel)
Source: Clin Rheumatol. 2022 Jan 1;41(4):1045–55. doi: 10.1007/s10067-021-05908-z (PMC8913559; doi:10.1007/s10067-021-05908-z)
Supplement: Supplementary file 1 — Supplementary file1 (PDF 484 KB) [file 10067_2021_5908_MOESM1_ESM.pdf]

Clinical Rheumatology

Supplementary Material

Effect of dose adjustments on the efficacy and safety of tofacitinib in patients with rheumatoid arthritis: a post hoc analysis of an open-label, long-term extension study (ORAL Sequel)

Ruediger B Mueller[1,2,3,*], Hendrik Schulze-Koops[3], Daniel E Furst[4], Stanley B Cohen[5], Kenneth Kwok[6], Lisy Wang[7], Tim Killeen[8], and Johannes von Kempis[2]

[1]*Rheumazentrum Ostschweiz, St. Gallen, Switzerland.* [2]*Division of Rheumatology and Immunology, Kantonsspital St. Gallen, St. Gallen, Switzerland.* [3]*Division of Rheumatology and Clinical Immunology, Department of Internal Medicine IV, Ludwig-Maximilians-University Munich, Munich, Germany.* [4]*UCLA, Los Angeles, CA, USA, University of Washington, Seattle, WA, USA, and University of Florence, Florence, Italy.* [5]*Metroplex Clinical Research Center and University of Texas Southwestern Medical Center, Dallas, TX, USA.* [6]*Pfizer Inc, New York, NY, USA.* [7]*Pfizer Inc, Groton, CT, USA.* [8]*Pfizer AG, Zürich, Switzerland.*

*Correspondence: PD Dr. Ruediger B Mueller, MD, MBA, Rheumazentrum Ostschweiz, 9000 St. Gallen, St. Jakobsstr. 20, Switzerland. Tel: +41 79 5062008.

E-mail: ruediger.mueller@hin.ch. ORCID iD: https://orcid.org/0000-0003-1997-5564.

Supplementary Table 1 Permitted reasons for tofacitinib dose adjustment

**5 → 10 mg BID**	**10 → 5 mg BID**
The investigator felt that the patient's rheumatoid arthritis symptoms were not adequately controlled on the lower dose, and the patient was not experiencing any tofacitinib-related AEs or laboratory abnormalities	Experience of AEs or laboratory abnormalities per-protocol, or at the investigator's discretion

AE adverse event, *BID* twice daily

Supplementary Table 2 Proportions of patients with missing data by efficacy outcome

Patients with missing data, n (%)	Dose-up (N=280)					Stay-on 5 (N=757)					Dose-down (N=476)					Stay-on 10 (N=2440)				
	Months after baseline					Months after baseline					Months after baseline					Months after baseline				
	**0**	**3**	**6**	**9**	**12**	**0**	**3**	**6**	**9**	**12**	**0**	**3**	**6**	**9**	**12**	**0**	**3**	**6**	**9**	**12**
ΔDAS28	1 (<1)	59 (21)	67 (24)	87 (31)	106 (38)	29 (4)	46 (6)	71 (9)	94 (12)	124 (16)	0 (0)	182 (38)	191 (40)	229 (48)	263 (55)	32 (1)	93 (4)	191 (8)	314 (13)	402 (16)
DAS28 MCID	-	59 (21)	67 (24)	87 (31)	106 (38)	-	46 (6)	71 (9)	94 (12)	124 (16)	-	-	-	-	-	-	-	-	-	-
DAS28 remission	-	59 (21)	67 (24)	87 (31)	106 (38)	-	30 (4)	48 (6)	71 (9)	108 (14)	-	182 (38)	191 (40)	229 (48)	263 (55)	-	66 (3)	165 (7)	291 (12)	376 (15)
DAS28 LDA	-	59 (21)	67 (24)	87 (31)	106 (38)	-	30 (4)	48 (6)	71 (9)	108 (14)	-	182 (38)	191 (40)	229 (48)	263 (55)	-	66 (3)	165 (7)	291 (12)	376 (15)
ΔHAQ-DI	1 (<1)	55 (20)	63 (23)	81 (29)	100 (36)	4 (<1)	15 (2)	47 (6)	75 (10)	103 (14)	0 (0)	180 (38)	187 (39)	228 (48)	262 (55)	21 (<1)	72 (3)	172 (7)	287 (12)	378 (15)

ΔCDAI	1 (<1)	55 (20)	65 (23)	86 (31)	104 (37)	4 (<1)	19 (3)	48 (6)	73 (10)	108 (14)	0 (0)	177 (37)	188 (39)	228 (48)	265 (56)	25 (1)	79 (3)	175 (7)	292 (12)	385 (16)
CDAI remission	-	55 (20)	65 (23)	86 (31)	104 (37)	-	16 (2)	45 (6)	70 (9)	105 (14)	-	177 (37)	188 (39)	228 (48)	265 (56)	-	59 (2)	158 (6)	276 (11)	368 (15)
ΔSDAI	1 (<1)	56 (20)	66 (24)	86 (31)	105 (38)	14 (2)	39 (5)	62 (8)	89 (12)	122 (16)	0 (0)	178 (37)	189 (40)	228 (48)	265 (56)	56 (2)	125 (5)	212 (9)	331 (14)	419 (17)
SDAI remission	-	56 (20)	66 (24)	86 (31)	105 (38)	-	28 (4)	52 (7)	79 (10)	113 (15)	-	178 (37)	189 (40)	228 (48)	265 (56)	-	78 (3)	166 (7)	290 (12)	378 (15)

Δ change from baseline, *CDAI* Clinical Disease Activity Index, *DAS28* 4-component Disease Activity Score in 28 joints, erythrocyte sedimentation rate, *HAQ-DI* Health Assessment Questionnaire-Disability Index, *LDA* low disease activity, *MCID* minimum clinically important difference, *N* number of patients in treatment group, *n* number of patients with missing data, *SDAI* Simplified Disease Activity Index

Supplementary Table 3 Summary of all-causality AEs: data at Months 3–6 and 6–12

AEs by selected time points[a]	Dose-up[b] (N=280)		Stay-on 5[c] (N=757)		Dose-down[b] (N=476)		Stay-on 10[c] (N=2440)	
	**Months 3–6**	**Months 6–12**	**Months 3–6**	**Months 6–12**	**Months 3–6**	**Months 6–12**	**Months 3–6**	**Months 6–12**
Evaluable for AEs, n	253	222	741	712	349	287	2377	2274
Total exposure, years	57	99	178	338	76	119	572	1061
Patients with AEs, n (%)	73 (28.9)	102 (45.9)	204 (27.5)	277 (38.9)	111 (31.8)	140 (48.8)	786 (33.1)	1128 (49.6)
Patients who discontinued due to AEs, n (%)	3 (1.2)	9 (4.1)	12 (1.6)	23 (3.2)	11 (3.2)	7 (2.4)	54 (2.3)	91 (4.0)
Most common AEs by preferred term, n (EAER per 100 patient-years)[d]								
Arthralgia	1 (1.75)	3 (3.04)	1 (0.56)	8 (2.36)	5 (6.53)	2 (1.68)	17 (2.97)	33 (3.11)
Blood creatine phosphokinase increased	2 (3.51)	6 (6.08)	3 (1.68)	6 (1.77)	3 (3.92)	5 (4.21)	16 (2.79)	23 (2.16)
Bronchitis	5 (8.79)	8 (8.11)	6 (3.37)	14 (4.13)	2 (2.61)	5 (4.21)	37 (6.47)	54 (5.08)
Hypertension	3 (5.27)	6 (6.08)	7 (3.93)	9 (2.66)	1 (1.30)	3 (2.52)	15 (2.62)	33 (3.11)
Nasopharyngitis	2 (3.51)	6 (6.08)	10 (5.62)	13 (3.84)	4 (5.22)	8 (6.74)	40 (6.99)	69 (6.50)
Rheumatoid arthritis[e]	4 (7.03)	4 (4.05)	4 (2.24)	4 (1.18)	2 (2.61)	7 (5.90)	15 (2.62)	35 (3.29)
Upper respiratory tract infection	3 (5.27)	7 (7.09)	14 (7.87)	18 (5.32)	11 (14.38)	13 (10.95)	51 (8.92)	78 (7.35)

Urinary tract infection	1 (1.75)	9 (9.12)	8 (4.49)	8 (2.36)	3 (3.92)	11 (9.27)	35 (6.12)	59 (5.56)
Investigations (SOC), n (EAER per 100 patient-years)	7 (12.31)	14 (14.19)	26 (14.61)	41 (12.11)	18 (23.53)	20 (16.85)	72 (12.59)	105 (9.89)
Selected investigations by higher-level group term, n (EAER per 100 patient-years)								
Hepatobiliary investigations	3 (5.27)	4 (4.05)	6 (3.37)	13 (3.84)	7 (9.15)	7 (5.90)	25 (4.37)	33 (3.11)
Renal and urinary tract investigations and urinalyses	0	2 (2.02)	3 (1.68)	5 (1.47)	5 (6.53)	0	7 (1.22)	7 (0.65)

[a]The reporting period (3–6 months or 6–12 months) is based on analysis baseline as 0 months

[b]For Dose-switch groups, analysis baseline was defined as the day of the (first) dose switch

[c]For Stay-on groups, analysis baseline was defined as LTE Month 3 visit

[d]AEs are presented for each dose group where EAER >6 in any dose group in Months 3–6 and 6–12

[e]All AEs coded as ‘rheumatoid arthritis’ in ORAL Sequel, except one, indicated worsening of rheumatoid arthritis

AE adverse event, *EAER* exposure-adjusted event rate per 100 patient-years, *LTE* long-term extension, *N* number of patients in treatment group, *n* number of patients with event, *SOC* system organ class

Supplementary Table 4 Summary of all-causality AEs: sensitivity analysis including data for patients who experienced multiple dose switches

**AEs by selected time points[a]**	**Dose-up (N=280)**				**Dose-down (N=476)**			
	**Months 0–3**	**Months 3–6**	**Months 6–12**	**Months >12**	**Months 0–3**	**Months 3–6**	**Months 6–12**	**Months >12**
Evaluable for AEs, n	280	268	252	223	476	431	392	321
Total exposure, years	68	64	116	573	113	101	173	523
Patients with AEs, n (%)	95 (33.9)	82 (30.6)	126 (50.0)	179 (80.3)	199 (41.8)	154 (35.7)	202 (51.5)	227 (70.7)
Patients who discontinued due to AEs, n (%)	5 (1.8)	3 (1.1)	12 (4.8)	30 (13.5)	19 (4.0)	13 (3.0)	14 (3.6)	36 (11.2)
Most common AEs by preferred term, n (EAER per 100 patient-years)[b]								
Blood creatine phosphokinase increased	2 (2.94)	3 (4.72)	7 (6.02)	14 (2.44)	7 (6.20)	3 (2.97)	6 (3.46)	16 (3.06)
Bronchitis	6 (8.83)	5 (7.87)	11 (9.46)	22 (3.84)	4 (3.54)	5 (4.95)	5 (2.88)	18 (3.44)

Herpes zoster	0	2	2	12	4	1	12	11
		(3.14)	(1.72)	(2.09)	(3.54)	(0.99)	(6.93)	(2.10)
Hypertension	3	3	7	21	4	3	4	13
	(4.41)	(4.72)	(6.02)	(3.66)	(3.54)	(2.97)	(2.31)	(2.48)
Nasopharyngitis	3	2	6	19	10	8	11	30
	(4.41)	(3.14)	(5.16)	(3.31)	(8.86)	(7.93)	(6.35)	(5.73)
Rheumatoid arthritis[c]	8	4	8	29	12	3	8	27
	(11.77)	(6.29)	(6.88)	(5.06)	(10.63)	(2.97)	(4.62)	(5.16)
Sinusitis	5	2	2	10	3	3	6	8
	(7.36)	(3.14)	(1.72)	(1.74)	(2.65)	(2.97)	(3.46)	(1.53)
Upper respiratory tract infection	3	3	10	21	10	14	19	42
	(4.41)	(4.72)	(8.60)	(3.66)	(8.86)	(13.88)	(10.97)	(8.03)
Urinary tract infection	5	1	11	21	6	6	16	18
	(7.36)	(1.57)	(9.46)	(3.66)	(5.31)	(5.95)	(9.24)	(3.44)
Investigations (SOC),	11	8	18	56	29	24	28	50
n (EAER per 100 patient-years)	(16.19)	(12.59)	(15.48)	(9.77)	(25.70)	(23.80)	(16.17)	(9.56)

Select investigations by higher-level group term, n (EAER per 100 patient-years)								
Hepatobiliary investigations	4 (5.88)	3 (4.72)	6 (5.16)	21 (3.66)	9 (7.97)	10 (9.91)	10 (5.77)	14 (2.67)
Renal and urinary tract investigations and urinalyses	2 (2.94)	0	2 (1.72)	12 (2.09)	8 (7.09)	6 (5.95)	1 (0.57)	7 (1.33)
AEs in overall period								
Patients with serious AEs, n (%) [EAER per 100 patient-years]	68 (24.3) [8.3]				92 (19.3) [10.1]			
AEs of special interest, IR per 100 patient-years (95% CI) [n (%)]								
Herpes zoster	1.94 (1.11–3.15) [16 (5.7)]				3.26 (2.20–4.66) [30 (6.3)]			
Serious infections	2.02 (1.18–3.24) [17 (6.1)]				2.84 (1.87–4.13) [27 (5.7)]			

Deep vein thrombosis	0.24 (0.03–0.86) [2 (0.7)]	0.10 (0.00–0.58) [1 (0.2)]
Pulmonary embolism	0.12 (0.00–0.66) [1 (0.4)]	0.21 (0.03–0.75) [2 (0.4)]

Analysis baseline was defined as the day of the (first) dose switch; as a sensitivity analysis, data beyond second dose switch are included for patients with multiple dose switches

[a]The reporting period (0–3 months, 3–6 months, 6–12 months, or >12 months) is based on analysis baseline as 0 months

[b]AEs are presented for each dose group where EAER >6 in any dose group at Months 0–3, 3–6, 6–12, or >12

[c]All AEs coded as ‘rheumatoid arthritis’ in ORAL Sequel, except one, indicated worsening of rheumatoid arthritis

AE adverse event, *CI* confidence interval, *EAER* exposure-adjusted event rate per 100 patient-years, *IR* incidence rate, N, number of patients in treatment group; *n* number of patients with event; *SOC* system organ class

Supplementary Fig. 1 a ΔCDAI, **b** CDAI remission, **c** ΔSDAI, and **d** SDAI remission over 12 months

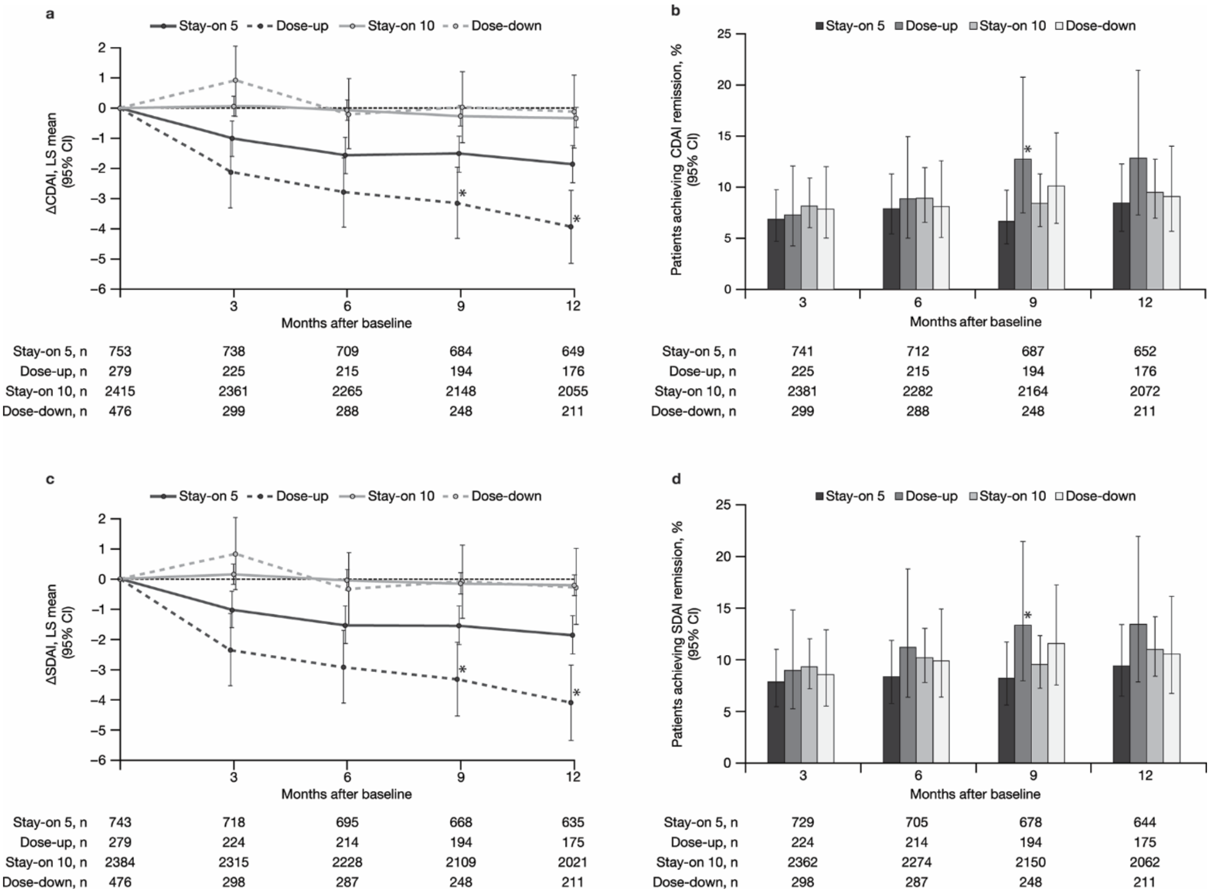

*$p<0.05$, Dose-up vs Stay-on 5

Δ change from baseline, *CI* confidence interval, *CDAI* Clinical Disease Activity Index, *LS* least squares, *n* number of evaluable patients, *SDAI* Simplified Disease Activity Index
